# Natural Killer T cells promote cholestatic liver disease independent of antigen presentation by cholangiocytes

**DOI:** 10.1097/HC9.0000000000000862

**Published:** 2026-01-16

**Authors:** Laura Valestrand, Kathrine S. Nordhus, Thierry Claudel, Jonas Øgaard, Anna Frank, Enya Amundsen-Isaksen, Claudia Fuchs, Natalie L. Berntsen, Brian K. Chung, Xiaojun Jiang, Michael Trauner, Espen Melum

**Affiliations:** 1 Norwegian PSC Research Center, Department of Transplantation Medicine, Division of Surgery and Specialized Medicine, Oslo University Hospital Rikshospitalet, Oslo, Norway; 2 Research Institute of Internal Medicine, Division of Surgery and Specialized Medicine, Oslo University Hospital Rikshospitalet, Oslo, Norway; 3 Institute of Clinical Medicine, Faculty of Medicine, University of Oslo, Oslo, Norway; 4 Section of Gastroenterology, Department of Transplantation Medicine, Division of Surgery and Specialized Medicine, Oslo University Hospital Rikshospitalet, Oslo, Norway; 5 Hans Popper Laboratory of Experimental Hepatology, Division of Gastroenterology and Hepatology, Department of Internal Medicine III, Medical University of Vienna, Vienna, Austria; 6 Hybrid Technology Hub-Centre of Excellence, Institute of Basic Medical Sciences, Faculty of Medicine, University of Oslo, Oslo, Norway

**Keywords:** ASBTcre, CD1d, cholangiocytes, cholestasis, NKT

## Abstract

**Background:**

Natural killer T (NKT) cells are an important lymphocyte subset in the liver that can be activated by CD1d-restricted lipid antigens presented on cholangiocytes, including lipids in bile from patients with chronic liver diseases. We hypothesized that during cholestasis, NKT cells play an essential role in the pathophysiology.

**Methods:**

We explored this immune pathway by ligating the common bile duct (BDL) in CD1d knockout (*Cd1d* ^−/−^) mice, *Traj18* ^*−/−*^ mice, wild-type (WT) mice, and created a novel model with conditional deletion of CD1d on cholangiocytes (*Cd1d* ^*ΔCC*^ mice).

**Results:**

Following BDL, we observed a clear and specific activation of hepatic NKT cells. *Cd1d* ^−/−^ mice, depleted of both invariant and noninvariant NKT cells, were protected from BDL-induced cholestatic liver damage. In contrast, *Traj18* ^*−/−*^ mice, lacking only invariant natural killer T cells, were not protected as compared with WT mice. *Cd1d* ^*ΔCC*^ mice exhibited a deficiency in functional CD1d expression on cholangiocytes but had a comparable distribution and phenotype of NKT cells as WT mice. After BDL, *Cd1d* ^*ΔCC*^ mice were not protected and developed similar liver disease as WT mice.

**Conclusions:**

A subset of hepatic NKT cells contributes to cholestatic liver disease, and their activation is independent of CD1d antigen presentation on cholangiocytes.

## Graphical abstract

**Figure:**
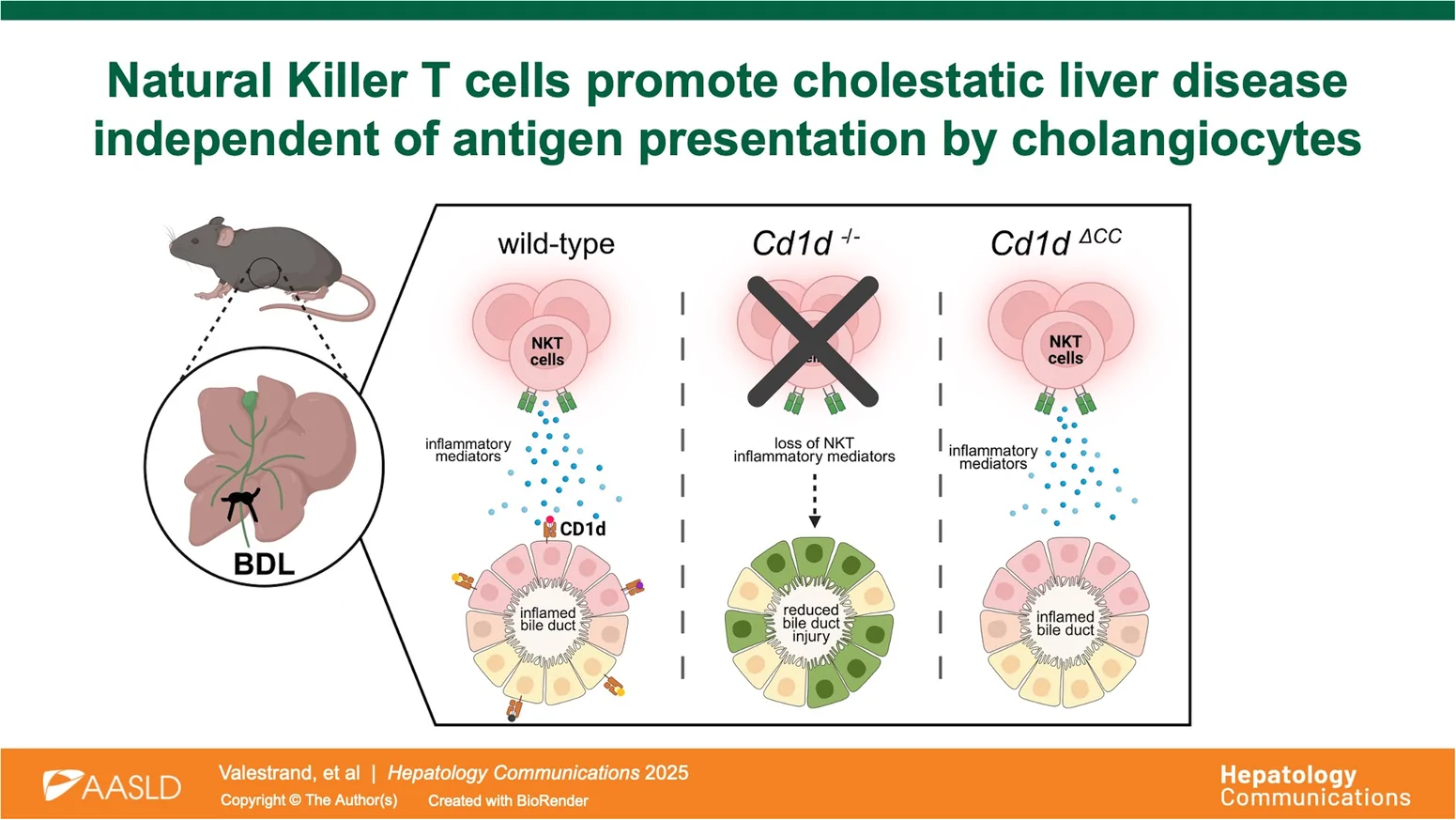

## INTRODUCTION

Cholestasis with retention of bile acids and other biliary constituents is a characteristic of a wide spectrum of liver diseases and can be the result of genetic, inflammatory, structural, and toxic mechanisms.^1^ The accumulation of intrahepatic bile acids leads to inflammation, bile duct proliferation, hepatocyte death, and eventually fibrosis and the development of cirrhosis if unresolved.^2^ This is exemplified by the inflammatory bile duct disease primary sclerosing cholangitis (PSC), where multiple fibroinflammatory strictures in the intrahepatic and extrahepatic bile ducts result in cholestasis and eventually cirrhosis with liver transplantation as the only curative treatment.^3^

The pathogenic process from impaired bile flow to cirrhosis is yet to be fully understood. In the widely used murine model of cholestasis with ligation of the common bile duct,^4^ bile canaliculi increase in diameter and the hepatic apical membrane and tight junctions are found to rupture with bile leaking to the sinusoids. This can again result in cell necrosis and the formation of nuclear fragmentation with rapid infiltration of neutrophil granulocytes which contribute to the formation of bile infarcts.^5^ Except in the setting of bile infarcts in bile duct–ligated mice, bile acids are not found to reach toxic levels that can cause direct necrosis of cells in cholestasis, and are more likely to act as signaling molecules, inducing an inflammatory response.^6^ Immunogenic mechanisms can play both detrimental and protective roles in cholestasis. Neutrophil granulocytes rapidly infiltrate the liver tissue and contribute to hepatocyte death through potent reactive oxygen species (ROS) formation, as demonstrated in livers of patients with primary biliary cholangitis (PBC).^7^ Furthermore, murine studies have demonstrated that T-helper 17 cells can promote liver disease in cholestasis, while Kupffer cells and regulatory T cells may be protective.^8^^–^^10^

Natural killer T (NKT) cells belong to a group of unconventional T cells that exhibit characteristics and functions of both the adaptive and innate immune system.^11^ NKT cells are especially prevalent in the liver and respond to lipid antigens presented by CD1d, a nonclassical major histocompatibility complex class I molecule.^11^ There are 2 main subclasses of NKT cells, invariant NKT (iNKT) cells expressing an invariant α-chain in combination with a restricted set of β-chains and noninvariant NKT (niNKT) cells harboring a more diverse set of T-cell receptors (TCRs).^11^ We have previously demonstrated that CD1d is expressed on the biliary epithelium and can present antigens to NKT cells^12^ and that injection of an NKT activating antigen directly in the bile ducts of mice results in activation of hepatic NKT cells.^13^ Both iNKT cells and niNKT cells can be activated by an array of antigens present in bile from patients with chronic liver diseases.^14^ Given these functions, NKT cells could potentially guide the direction of an immune response in cholestasis with their rapid cytokine production and the ability to engage downstream immune subsets.^11^ The immune pathway involving bile and NKT cells may represent a mechanistical link of relevance for the clear association between patients with PSC and inflammatory bowel disease, and between chronic liver diseases and gut dysbiosis.^15^ Indeed, NKT cell levels and functions have been linked to a range of liver diseases and promote disease in murine models of PBC-like disease,^16^ autoimmune hepatitis mediated by IFN-γ,^17^ in steatohepatitis-associated hepatocellular carcinoma^18^ and in a hepatic inflammatory model with intrabiliary injection of oxazolone.^13^ In contrast, they are also suggested to protect against liver disease, as in an hepatitis B virus infection model, where they reinforce the anti-viral T-cell and B-cell immune response.^19^ In the BDL model in mice, iNKT cells are suggested to protect against cholestatic disease.^10^^,^^20^ Cholangiocytes express functional CD1d^12^ with expression levels varying with disease stage in PBC, PSC, alcohol-associated liver disease and biliary atresia.^12^^,^^21^ Cholangiocytes also have immunomodulatory properties in both adaptive and innate immune responses^22^ and are continuously exposed to lipid-rich bile with a potential role in NKT-mediated inflammation.

To clarify the exact role of NKT cells during cholestasis, we performed BDL to model cholestasis and investigated the role of NKT cells during cholestasis in both *Cd1d* ^*−/−*^ mice that lack CD1d and all NKT cell subgroups, and in *Traj18* ^*−/−*^ mice lacking only iNKT cells. Then, to explore the role of cholangiocytes as antigen-presenting cells (APCs) in NKT cell–related inflammation, we generated a novel conditional knockout mouse model lacking CD1d on the biliary epithelium (*Cd1d* ^*ΔCC*^).

## METHODS

### Human samples

Fresh-frozen liver biopsies from patients with PSC and disease controls were previously collected and analyzed by single-nuclei RNA-sequencing as described.^23^ These data can be accessed at https://riim-data.no/NoPSC_Liver_Atlas/SINGLE_NUC/. Written consent was obtained from all study participants before sampling, and study ethics were approved by the regional Committees for Medical and Health Research Ethics of Southeast Norway (reference numbers: 15368 and 599798) in accordance with the Declaration of Helsinki and Istanbul. Processing of the samples, single-nuclei RNA sequencing (snRNA-seq), and data analysis have been described^23^ (please see Supplemental Methods for details, http://links.lww.com/HC9/C206).

### Mice

All experimental mice were housed in a minimal disease unit at the animal facility at Oslo University Hospital Rikshospitalet, Oslo, Norway (please see Supplemental Methods, http://links.lww.com/HC9/C206 for details on mice strains). All experiments were performed using male or female mice aged 8–12 weeks at the time of surgery. They were not fasted, and the surgery was carried out during the light cycle. All animals received humane care and were kept with a 12-hour light-dark cycle with *ad libitum* access to water and standard rodent diet. The animal experiments were approved by the Norwegian National Animal Research Authority (project license no FOTS 8210 and FOTS 21918), the Animal Ethics Committee of the Medical University of Vienna, and the Federal Ministry of Science, Research and Economy (BMWFW-66.009/0146-WF/II/3b/2014) and were performed in accordance with the European Directive 210/63/EU, the Animal Research: Reporting of *In Vivo* Experiments guidelines and The Guide for the Care and Use of Laboratory Animals, eighth edition (NRC 2011, National Academic Press).

### Generation of *ASBTcre* ^+/−^ mice

A bacterial artificial chromosome (BAC) (RP23–178M8) containing the *Asbt* (*Slc10a2*) gene was modified via homologous recombination in *E. coli* (for details, please see the Supplemental Methods, http://links.lww.com/HC9/C206). The resulting BAC DNA was pronuclear-injected at 1 ng/μL in C57BL/6-derived oocytes. *ASBTcre* ^+/−^ mice were genotyped using the oligos: CTTTCTGTCTTGGCCAGTAGTTTCTCTGG and GCTTGCAGGTACAGGAGGTAG.

### Crossings of mice and genotyping

*ASBTcre* ^*+/*−^ mice carrying the *cre* gene in the *Asbt* promoter were bred as heterzygotes and genotyped at each round to confirm the heterozygous presence of the *cre* gene (see Supplemental Methods, http://links.lww.com/HC9/C206, for details). *Cd1d1* ^*fl/fl*^ mice were bred as homozygotes, and the presence of 2 alleles of *loxP* sites was confirmed by genotyping (see Supplemental Methods, http://links.lww.com/HC9/C206, for details). To specifically delete CD1d on the cholangiocytes, *ASBTcre* ^−/+^ mice were bred with *Cd1d1* ^*fl/fl*^ mice to obtain litters, including *ASBTcre* ^−/+^ *Cd1d1* ^fl/fl^ mice, hereafter named *Cd1d* ^*ΔCC*^. Please see Supplemental Methods, http://links.lww.com/HC9/C206, for details on the generation and breeding of the strain. In experiments, we used *Cd1d* ^*ΔCC*^ male and female, 8–12 weeks old mice with littermate *ASBTcre* ^−/−^ *Cd1d* ^*fl/fl*^ considered as and named WT controls.

### Bile duct ligation

Cholestasis was induced by ligation of the common bile duct as described.^24^ All procedures were performed with analgesia (bupreorphine 0.2 mg/kg) and under general anesthesia with either Isoflurane gas (1% for maintenance and 4% for introduction of anesthesia, in 100% oxygen, flow rate, 4 L/min) or intraperitoneal injection with 0.1 mg/g FD2 (fentanyl/domitor/dormicum) and 1 mg/kg Antisedan (antagonist) post-surgery. Mice were kept on a (37 °C) heating pad during the complete procedure, and their eyes were covered with eye ointment. The abdomen was sterilized using a gauze swab moistened with antiseptic, and after ensuring deep anesthetization, a 1.5–2 cm long midline upper abdominal incision was made. Peritoneum was cut in the linea alba, and the liver was lifted with a saline (0.9% NaCl solution) moistened cotton swab to reveal the hepatic hilar area. The common bile duct was identified, dissected free from the flanking portal vein and hepatic arteries and a simple ligation with a 6-0 suture (Medtronic) was placed in the proximal hilar area distal to the first bile duct branching. Before the closure of fascia and skin, the liver was repositioned to the correct anatomical position and loss of fluid by evaporation from the abdominal cavity was replaced with saline. Sham mice underwent an identical procedure as mice subjected to BDL, including dissection of the bile duct, but without the ligation. Evaluation of clinical condition was performed with a predefined score scheme, including evaluation of weight, appearance, behavior, and clinical signs. Postoperative analgesia was administered with buprenorphine (0.2 mg/kg).

### Mouse tissue collection and extraction of primary lymphocytes

On days 3 and 5 after BDL, blood and liver tissue were collected immediately after euthanization of the animal. Please see Supplemental Methods, http://links.lww.com/HC9/C206, for further details. To isolate primary lymphocytes for flow cytometry, livers were perfused at harvest with 10 mL of cold PBS before livers and spleens were collected in tubes with cold PBS. Details of the following extraction of lymphocytes are given in the Supplemental Methods, http://links.lww.com/HC9/C206.

### Isolation of cholangiocytes from murine livers

Mice were sacrificed by columnar neck stretch or a CO_2_ overdose, the abdomen was opened, and the liver and portal vein were exposed carefully. A 22G needle was inserted into the portal vein and the liver perfused with 20 mL of liver perfusion medium (Thermo Fisher Scientific) to remove the blood from the liver, followed by perfusion of 10 mL collagenase I solution (0.5 mg/mL). Next, the liver was isolated and washed several times with organoid wash medium, containing epidermal growth factor (EGF) and rho-kinase inhibitor Y-27632, and brought into a sterile cell culture bench. The liver surface was carefully scraped with a scalpel, thereby disrupting the liver tissue and revealing the biliary tree. To release small clumps of cholangiocytes from the biliary epithelial tissue, the biliary tree was transferred into a new petri dish filled with organoid wash-medium^25^ and then carefully scraped with a scalpel and cut into small pieces. Isolated cholangiocyte clumps and remaining biliary tissue were then mixed with Matrigel (1:3, one part triple-concentrated culture medium, 2 parts Matrigel) and seeded as 50 μL droplets into cell culture dishes.^25^

### Organoid cell culture

To increase the cholangiocyte cell number to confirm the conditional knockout of CD1d, we generated cholangiocyte organoids that display full functionality after cell culture. Cell culture of cholangiocyte organoids was performed as described.^25^ The exact composition of organoid wash and culture medium was similar, except for the use of murine, instead of human EGF,^25^ (please see Supplemental Methods, http://links.lww.com/HC9/C206, for details).

### Co-culture experiments with NKT cells

Cholangiocyte organoids from WT, *Cd1d* ^*−/−*^, and *Cd1d* ^*ΔCC*^ mice were mechanically disrupted into smaller cholangiocyte clumps and seeded as cholangiocyte single cells (100,000 cells/well) into conventional tissue culture plates (coated with a thick layer of collagen I, 2.5 mg/mL) in supplemented Williams E+ (Williams E+ EGF + Y-27632 + R-Spondin + DKK-1) for 48 hours. The presence of CD1d was evaluated by the secretion of NKT cell–derived IL-2 as a marker of activation, after co-culture with NKT-cell hybridomas and the potent iNKT cell–activating ligand α-galactosylceramide (see Supplemental Methods, http://links.lww.com/HC9/C206, for details).

### Flow cytometry

Single-cell suspensions of primary lymphocytes from spleens and livers were incubated with Fc-block (antibody details are given in Supplemental Table 1, http://links.lww.com/HC9/C206) for 30 minutes to avoid nonspecific binding. Followed by 1 hour dark incubation at 4 °C with monoclonal antibodies (detailed information is provided in Supplemental Table 1, http://links.lww.com/HC9/C206) and PE-labeled α-galactosylceramide–loaded PBS-57 CD1d tetramers (kindly provided by the NIH Tetramer Core). CountBright absolute counting beads (Thermo Fisher Scientific) were used to estimate the absolute cell count.

To analyze cholangiocyte organoids by flow cytometry, the cholangiocytes were first removed from Matrigel using mechanical disruption and Cell Recovery Solution (cat. Nr: 354253, Merck KGaA), collected in a 15 mL Falcon tube and incubated on ice for 30 minutes. The disrupted organoids were then washed twice with supplemented Williams E medium^25^ and dissociated to single cells using Trypsin/EDTA solution (cat. Nr: 25200-056, Life Technologies) for 8 minutes at 37 °C. The steps as described above for the flow cytometry procedure were undertaken, and cholangiocytes were stained with CK19 monoclonal antibody and CD1d monoclonal antibody. For intracellular staining, cells were fixed and permeabilized with Foxp3/Transcription Factor Staining Buffer Set (Thermo Fisher Scientific). Flow cytometric analysis was performed using a BD FACS Verse TM flow cytometer (BD Biosciences) or MACSQuant Analyzer (Miltenyi Biotec), and results were analyzed in FlowJo version 10.6.2

### Histology and assessment of cholangitis and necrosis

Formalin-fixed liver tissue was embedded in paraffin, cut into 3 μm sections and H&E-stained before they were scanned using an Axioscan Z1 (Zeiss, Carl-Zeiss Stiftung) and kept digitally in a local whole slide centralized storage library. Portal inflammation was evaluated in a blinded manner, and necrosis was assessed semiautomatically (see Supplemental Methods, http://links.lww.com/HC9/C206, for further details).

### Immunohistochemistry and immunofluorescent staining

Three micrometer sections of formalin-fixed and paraffin-embedded livers were stained for CD3^+^ cells or lymphocyte antigen 6 complex locus G6D (Ly6g^+^) cells. Details are given in Supplemental Methods, http://links.lww.com/HC9/C206. DAB-stained slides were treated and stored digitally as the HE slides. Cell detection was performed using default settings in QuPath v3.4.0. Within each experimental repeat, cells were labeled as positive, negative, or to be ignored based on morphological characteristics and staining intensity to remove unspecifically stained cells, and a dense neural network was trained to distinguish between the 3 categories. Labels were added until the model was performing with 95% specificity and accuracy or above, and lastly, cells in each category were counted and their numbers adjusted to the total cell population. Immunofluorescence staining was performed using TSA-amplification of CD1d signals as described previously by Berntsen et al.^13^ Details are given in Supplemental Methods, http://links.lww.com/HC9/C206.

### Single-nuclei RNA-sequencing of formalin-fixed paraffin-embedded liver blocks from mice

Liver sections (3×25 µm) from formalin-fixed paraffin-embedded (FFPE) blocks from 3 of the genotype *Traj18* ^−/−^, as well as from 4 WT mice, were prepared according to the 10X protocol for single-nuclei sample preparation from FFPE sections (CG000784)^26^ (please see Supplemental Methods, http://links.lww.com/HC9/C206, for details on processing and postsequencing analysis). These data can be accessed at https://riim-data.no/portal.html?dataset=BDL/NKT.

### Statistical analysis

All values are presented as mean ± SEM unless otherwise stated. Statistical significance was evaluated using Student *t* test for variables meeting the criteria of normal distribution. Mann-Whitney *U* test was used for variables not meeting the criteria of normal distribution. *p* values below 0.05 were considered statistically significant. For the snRNA-seq, statistical significance (*p*<0.05) of indicated genes in NKT cells was calculated using the Benjamini-Hochberg correction for multiple testing in the Pseudobulk function of Loupe browser v.7.0.1 (10X Genomics). All other statistical tests were performed using GraphPad Prism v.10.0.3 and 8.2 (GraphPad Software).

## RESULTS

### Specific activation of iNKT cells

To investigate immune regulation during cholestasis, we ligated the common bile duct in WT mice and used sham-operated mice as controls. After 3 and 5 days, representing the acute and intermediate phase after BDL, hepatic and splenic lymphocytes were isolated and subjected to flow cytometry analysis (gating strategies are shown in Figure 1A). At day 3 after BDL, hepatic iNKT cells had an activated phenotype with increased CD69 and CD25 expression levels compared to hepatic iNKT cells from noncholestatic sham-operated mice (Figure 1B), implying that iNKT cells have a specific role in the pathogenesis. There was a significant apparent reduction in the absolute number of iNKT cells (Figure 1C) in cholestatic mice, which can indicate NKT cell activation as the TCR has been shown to be downregulated on activation.^27^ The activated iNKT cells had a slight reduction in CD4^+^ cells and an increase in the double-negative compartment (Figure 1C). The activation in the acute phase was persistent to day 5 after BDL (Figure 1D) when recruitment of nontissue resident immune subsets, such as circulating TCRβ^+^cells, could be expected. The TCRβ^+^iNKT^−^ population was reduced in the initial phase, 3 days after BDL, but was not activated neither in the initial nor the intermediate setting, 5 days after BDL (Figures 1E–G), suggesting NKT-specific activation. In accordance with previous studies using the BDL model, we observed recruitment of Ly6G^+^ neutrophil granulocytes and TCRβ^−^NK1.1^+^ cells at both day three and day 5 after BDL (Figures 1H–J).^10^^,^^20^ There were no observed changes for CD19^+^ cells (Figures 1I, J). Peripheral splenic iNKT cells were also activated in bile duct–ligated mice in comparison to sham-operated mice, indicating systemic activation or circulation of activated NKT cells (Supplemental Figures 1A and B, http://links.lww.com/HC9/C206). In contrast to the inactivated liver TCRβ^+^iNKT^−^ cells, the peripheral splenic TCRβ^+^iNKT^−^ population was activated at day 3 with upregulation of CD25 (Supplemental Figure S1A, http://links.lww.com/HC9/C206), but not at day 5 after BDL (Supplemental Figure S1B, http://links.lww.com/HC9/C206), reflecting a systemic immediate T-cell immune response after BDL.

**FIGURE 1:**
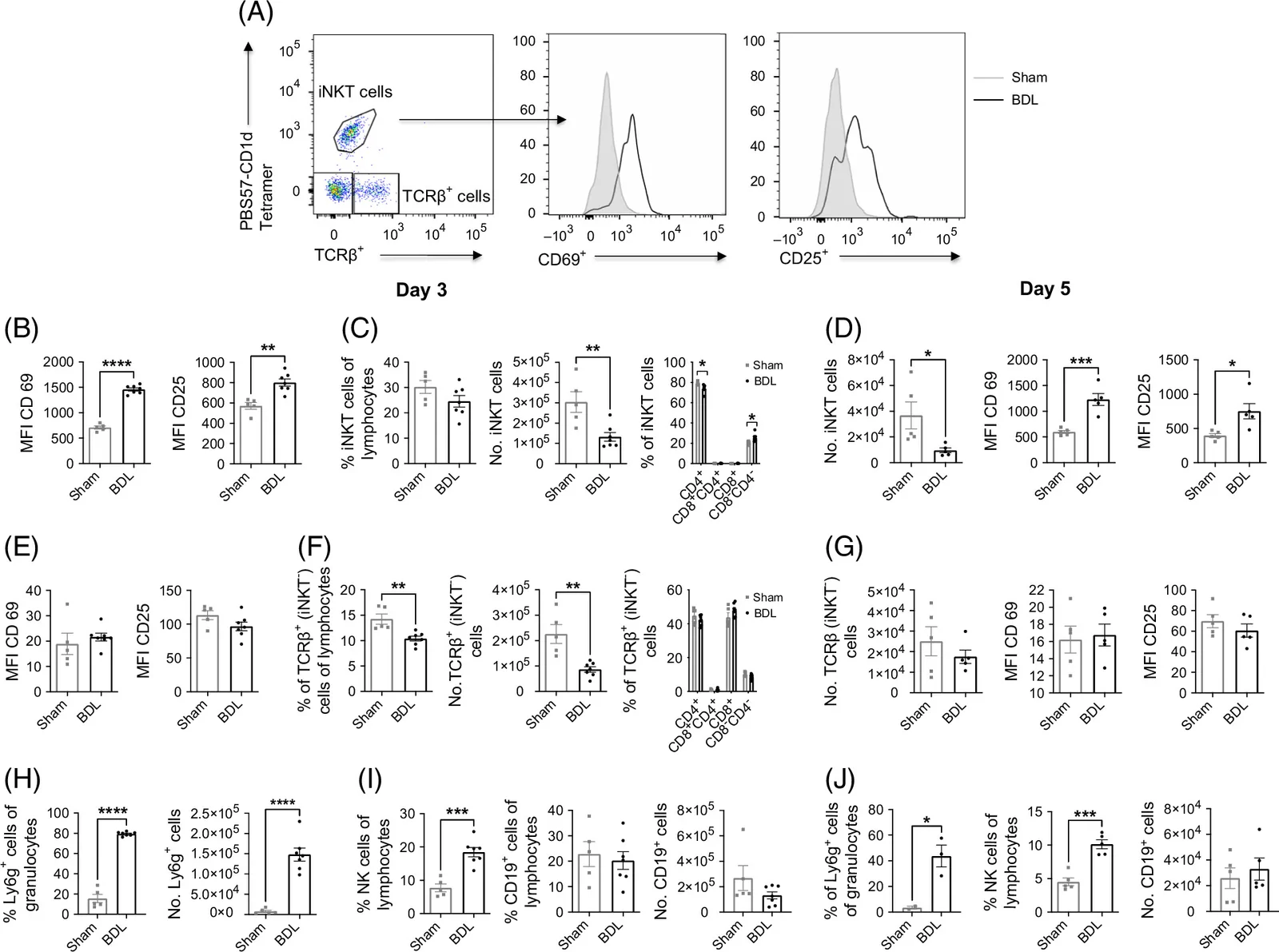
Specific activation of iNKT cells in bile duct–ligated mice. (A) Representative plots with the flow gating strategies for iNKT cells, defined as teteramer^+^TCRβ^+^ cells, and for tetramer^-^TCRβ^+^ cells in mononuclear cell suspension isolated from livers, 3 days after BDL. Representative histograms with MFI of the activation markers CD69 and CD25 on iNKT cells. Black lines represent BDL mice, and gray histograms represent sham-operated mice. (B) Bargraphs showing the MFI of the activation markers CD69 and CD25 on iNKT cells 3 days after BDL. (C) The percentage and absolute number of iNKT cells 3 days after BDL, and CD4/CD8 distribution of the iNKT cells. (D) The absolute number of iNKT cells and the MFI of the activation markers CD69 and CD25 on iNKT cells 5 days after BDL. (E) Bargraphs showing the MFI of the activation markers CD69 and CD25 on TCRβ^+^iNKT^−^cells 3 days after BDL. (F) The percentage and absolute number of of TCRβ^+^iNKT^-^ cells 3 days after BDL, and CD4/CD8 distribution of TCRβ^+^iNKT^-^ cells. (G) The absolute number of TCRβ^+^iNKT^−^ cells and the MFI of the activation markers CD69 and CD25 on TCRβ^+^iNKT^-^ 5 days after BDL. (H) Percentage and absolute number of Ly6g^+^ cells among granulocytes 3 days after BDL. (I) Percentage of TCRβ^−^NK1.1^+^ (NK) cells and CD19^+^ (B) cells, and absolute number of CD19^+^ cells 3 days after BDL. (J) Percentage of Ly6g^+^ among granulocytes, and NK and B-cells 5 days after BDL. (B-J) Experiments compare iNKT cells or TCRβ^+^iNKT^-^ cells from bile duct–ligated mice (n=7) compared to sham mice (n=5), 3 days after BDL in panel B,C,E,F,H, I or from bile duct–ligated mice (n=5) compared to sham mice (n=5) 5 days after BDL in panel D, G, J. All experiments are repeated 2 independent times, and results from one representative experiment are shown as mean ± SEM. Statistical significance was evaluated by a 2-tailed Student *t* test. * p<0.05, ** p<0.01, *** p<0.001, **** p<0.0001. Abbreviations: BDL, bile duct ligation; iNKT, invariant natural killer T; MFI, median fluorescence intensity; NK, natural killer; WT, wild type.

### NKT cells promote cholestatic liver injury

Having demonstrated specific activation of iNKT cells both in the acute and intermediate phase after BDL, we aimed to investigate their direct role in disease development. To do so, we ligated the common bile duct of *Cd1d* ^*−/−*^ mice globally deficient of CD1d and all subclasses of NKT cells and compared them to bile duct–ligated WT mice. The mice were monitored daily, and serum and livers were collected on day 3 after BDL. Already at day 1 after surgery, WT mice lost more weight than mice lacking CD1d and NKT cells, which persisted until the day of harvest at day 3 (Figure 2A, clinical score see Supplemental Figure S2, http://links.lww.com/HC9/C206). The cholestatic liver biochemistry profile was less severe in *Cd1d* ^*−/−*^ mice with lower levels of both bilirubin and ALP (Figure 2B), and the hepatocellular damage marker ALT showed a trend to lower levels (Figure 2B), supporting the clinical data of a milder disease course following BDL in *Cd1d* ^*−/−*^ mice. Histological examinations of livers from *Cd1d* ^*−/−*^ mice showed less necrosis and trending fewer infiltrating neutrophil granulocytes (Figures 2C, D). There was no difference in the infiltration of T cells or distribution of immune cells in portal areas when comparing *Cd1d* ^*−/−*^ and WT mice (Figures 2E, F). To clarify the contribution of different subsets of NKT cells, we next ligated the bile ducts of *Traj18* ^*−/−*^ mice that have intact CD1d expression but lack iNKT cells.^28^ We did not observe any differences in weight, liver biochemistry or liver histopathology between *Traj18* ^*−/−*^ mice and WT mice (Figures 3A–C). To investigate mechanisms driving the disease phenotype, we performed snRNA-seq of livers 3 days after BDL from *Traj18* ^*−/−*^ mice to characterize niNKT cells and in WT mice to characterize the complete NKT cell population. We defined NKT cells as *Klrb1c* ^+^/*Cd3e* ^+^, in line with the literature,^29^ and compared NKT cells between *Traj18* ^−/−^ mice and WT mice. NKT cells from both WT mice and *Traj18* ^−/−^ mice exhibited an activated phenotype (Supplemental Figure S3, http://links.lww.com/HC9/C206). Of note, the defined NKT cells in WT mice expressed IL4, (Supplemental Figure S3, http://links.lww.com/HC9/C206), while this cytokine was absent in *Traj18* ^*−/−*^ mice, since these mice exhibit the same clinical phenotype and response following BDL (Figure 3A-C), it can be speculated that this cytokine is not central in the pathogenesis. Taken together, these results indicate that NKT cells promote disease and that a subset of NKT cells drives the pathological process in bile duct–ligated mice. To assess if NKT cell activation during cholestasis was also relevant in humans, we assessed the phenotype of NKT cells by snRNA-seq in patients with PSC (n=12) and observed an activated phenotype of NKT cells with upregulation of CD69 and CD44 as compared to a noncholestatic disease control group (n=4) (Figures 3D–F). These findings are completely in line with the murine results and show a potential relevance for the NKT cell pathway in human cholestatic conditions.

**FIGURE 2:**
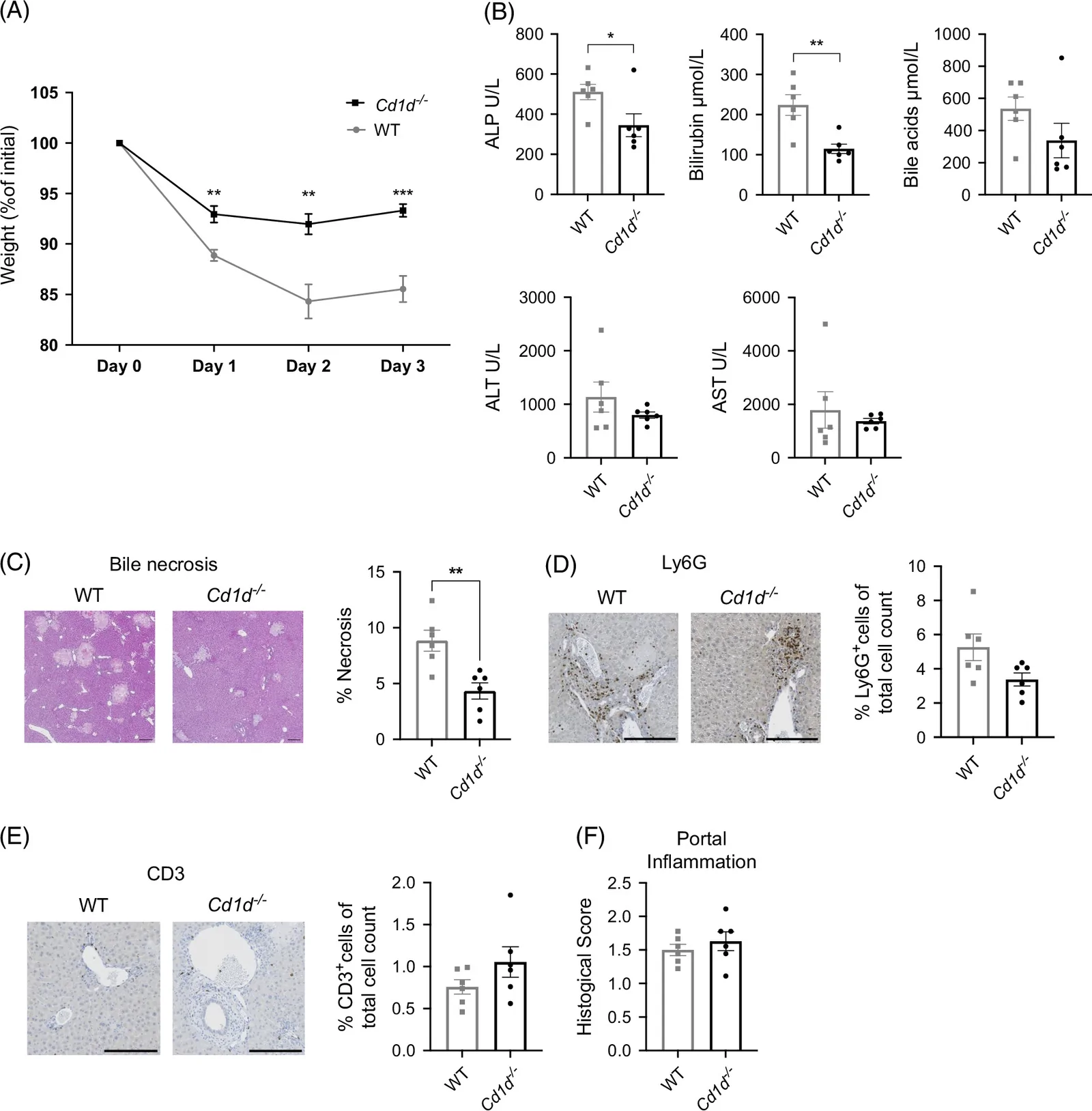
NKT cells promote cholestatic liver injury. (A) Weight curve comparing bile duct–ligated *Cd1d* ^*−/−*^ mice (n=6) and WT mice (n=6) 3 days after surgery. (B) Serum biochemistry at day 3 after BDL of *Cd1d* ^*−/−*^ mice and WT mice. (C) Representative images of hematoxylin and eosin–stained liver sections and semiautomated quantification of percentage of necrosis in *Cd1d* ^*−/−*^ and WT mice 3 days after BDL using an artificial neural network algorithm. (D, E) Representative images of DAB-stained liver sections and percentage of Ly6G^+^ and CD3^+^ cells per total cell count using an artificial neural network algorithm in *Cd1d* ^*−/−*^ and WT mice 3 days after BDL. (F) Assessment of portal inflammation in *Cd1d* ^*−/−*^ and WT mice 3 days after BDL by using a predefined histology score from 1 to 3. All experiments were repeated 3 times independently, and results from one representative experiment are shown as mean ± SEM. Statistical significance was evaluated by a 2-tailed Student *t* test. * p<0.05, ** p<0.01, *** p<0.001. Abbreviation: BDL, bile duct ligation; NKT, natural killer T; WT, wild type. Scale bar=200 µm

**FIGURE 3:**
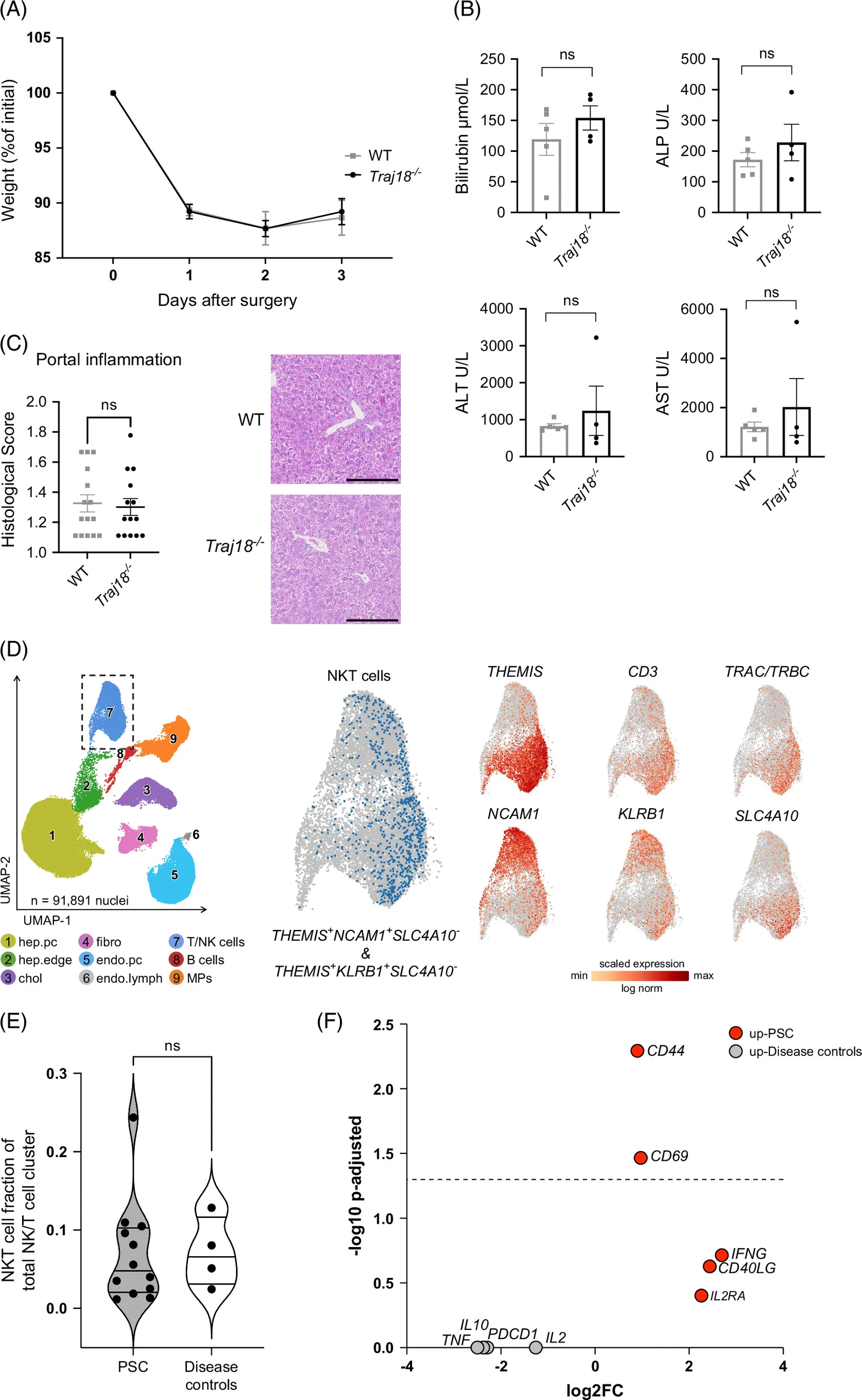
iNKT cells are not driving the disease development in bile duct–ligated mice, and NKT cells are activated in a human cholestatic condition. (A, B) Weight curve and serum biochemistry comparing bile duct–ligated *Traj18* ^*−/−*^ mice (n=4) and wild-type (WT=5) mice for 3 days after surgery. (C) Assessment of portal inflammation in *Traj18* ^*−/−*^ mice and WT mice 3 days after bile duct ligation by using a predefined histology score from 1 to 3 and representative images of hematoxylin and eosin–stained liver sections. (D) Characterization by single-nuclei sequencing of hepatic NKT cells, defined as (*THEMIS* ^*+*^ *NCAM1* ^+^ *SLC4A10* ^−^ or *THEMIS* ^*+*^ *KLRB1* ^+^ *SLC4A10* ^−^) in liver samples from patients with PSC (n=12) and noncholestatic disease controls (n=4). (E) Relative abundance of NKT cells between patients with PSC and disease controls. Statistical significance was tested by the Mann-Whitney *U* test. (F) Volcano plot demonstrating upregulated activation markers CD69 (*p*=0.016) and CD44 (*p*=0.025) in NKT cells from patients with PSC compared to disease controls. All murine experiments were repeated 3 times independently, and the results from one representative experiment are shown as mean ± SEM. For the SnSeq data, the statistical significance (*p*<0.05) of indicated genes in NKT cells was calculated using the Benjamini-Hochberg correction for multiple testing in the Pseudobulk function of Loupe browser v.7.0.1 (10x Genomics). For all other analyses, statistical significance was evaluated by a 2-tailed Student *t* test. Abbreviations: NKT, natural killer T; PSC, primary sclerosing cholangitis; WT, wild type. Scale bar=200 µm

### NKT cell–mediated pathology is not driven by cholangiocytes

Since cholangiocytes express functional CD1d^12^ and have immunomodulatory properties in both adaptive and innate immune responses,^22^ we examined the specific role of CD1d on cholangiocytes during cholestasis. We generated a novel mouse model with conditional knock out of CD1d on the biliary epithelium by crossing *Cd1d1* ^*fl/fl*^ mice with mice expressing *Cre* controlled by the *Asbt* promotor. In short, a BAC (RP23–178M8) containing the *Asbt* gene (*Slc10a*) was modified via homologous recombination in *E.coli* ^30^ with a cassette containing *Cre* (Figure 4A). This was followed by pronuclear injection of recombinant BAC DNA into C57BL/6-derived oocytes and transferred to pseudo-pregnant foster mice. The *ASBTcre* ^*+/*−^ mice were genotyped to confirm the presence of *Cre*, where a 4-kb band in gels represents the WT allele and a 1.4-kb band represents the recombinant allele (Figure 4B). The co-expression of *Cre* with *Asbt* results in conditional knockout of CD1d in large cholangiocytes, gallbladder-derived cholangiocytes, enterocytes in the terminal ileum and in the proximal renal tubule cells.^31^^,^^32^ The phenotype of the cholangiocytes in these mice was established by first demonstrating a lack of CD1d expression on cholangiocyte organoids derived from *Cd1d* ^*−/−*^, and *Cd1d* ^*ΔCC*^ compared to WT mice (Figure 4C). By using co-culture experiments with cholangiocyte organoids and an iNKT cell hybridoma, we confirmed that this loss of CD1d was functional and not due to CD1d internalization, as α-galactosylceramide–loaded cholangiocyte organoids generated from *Cd1d* ^*−/−*^ and *Cd1d* ^*ΔCC*^ mice did not elicit IL2 production by the hybridoma. Cholangiocyte organoids from WT mice could activate the NKT cell hybridoma, as evidenced by IL-2 production (Figure 4D). Conditional knockout of *Cd1d1* on cholangiocytes did not affect iNKT cell development compared to WT mice, and no perturbations were observed in the other immune subsets (TCRβ^+^ cells, CD19^+^cells, or natural killer cells) examined in the liver and spleen (Figure 4E). After having confirmed the phenotype and demonstrated a normal immune distribution following conditional deletion of *Cd1d1*, we investigated the role of cholangiocytes as APCs during cholestasis by ligating the bile ducts of *Cd1d* ^*ΔCC*^ and *Cd1d1* ^*fl/fl*^ mice. With the flanking *loxP* sites to *Cd1d1* being the only difference to WT mice, we used *Cd1d1* ^*fl/fl*^ as controls. There were no differences in weight loss, liver biochemistry, bile infarcts, or portal inflammation between the *Cd1d* ^*ΔCC*^ and WT mice after BDL (Figures 5A–C), reflecting that cholangiocytes are not acting as the main APC engaging NKT cells in the pathological process leading to cholestatic liver damage. Immunofluorescent staining and snRNA-seq characterization of CD1d in the cholestatic livers (Supplemental Figure S4, http://links.lww.com/HC9/C206) highlight other possible APC candidates.

**FIGURE 4:**
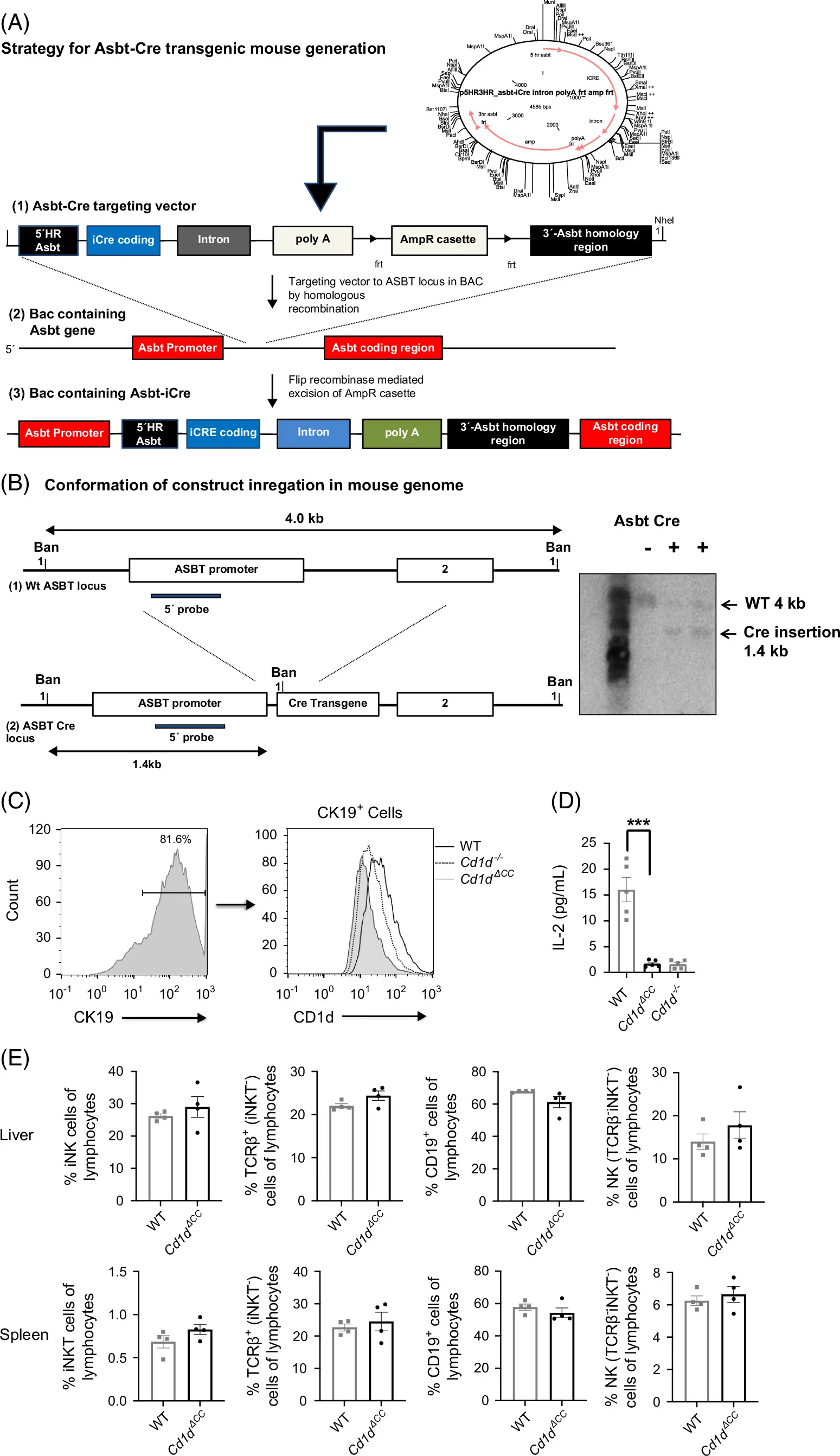
A novel mouse model with conditional knockout of CD1d on biliary epithelium. (A) Schematic overview of the construct of the BAC and strategy for *Asbt*-*Cre* transgenic mouse generation. (B) Confirmation of the construct in the mouse genome by genotyping. (C) Representative plot showing count of CK19^+^ cells among cholangiocyte organoids and flow plots with CD1d expression on cholangiocyte organoids derived from WT (black line), *Cd1d* ^*−/−*^ (black dotted line) and *Cd1d* ^*ΔCC*^ (light gray histograms) mice. (D) Co-culture experiment with WT-, *Cd1d* ^*−/−*^-, and *Cd1d* ^*ΔCC*^ *-*cholangiocyte organoids as antigen-presenting cells, alpha-galactocylseramide (αGalCer), a potent iNKT cell ligand and an invariant iNKT cell hybridoma (DN32.D3). Activation was measured as IL-2 concentration in the supernatant medium by ELISA. The experiment was repeated 2 times, and the results from one representative experiment are shown as mean ± SEM. Statistical significance was evaluated by a 2-tailed Student *t* test. (E) Immunophenotyping of iNKT^+^, TCRβ^+^iNKT^−^, CD19^+^, and NK^+^ cells by flow cytometry in spleen and liver of WT (*ASBTcre* ^−/−^ *Cd1d1* ^fl/fl^) (n=4) and *Cd1d* ^*ΔCC*^ mice (n=4). *** p<0.001. Abbreviations: BAC, bacterial artificial chromosome; iNKT, invariant natural killer T; WT, wild type.

**FIGURE 5:**
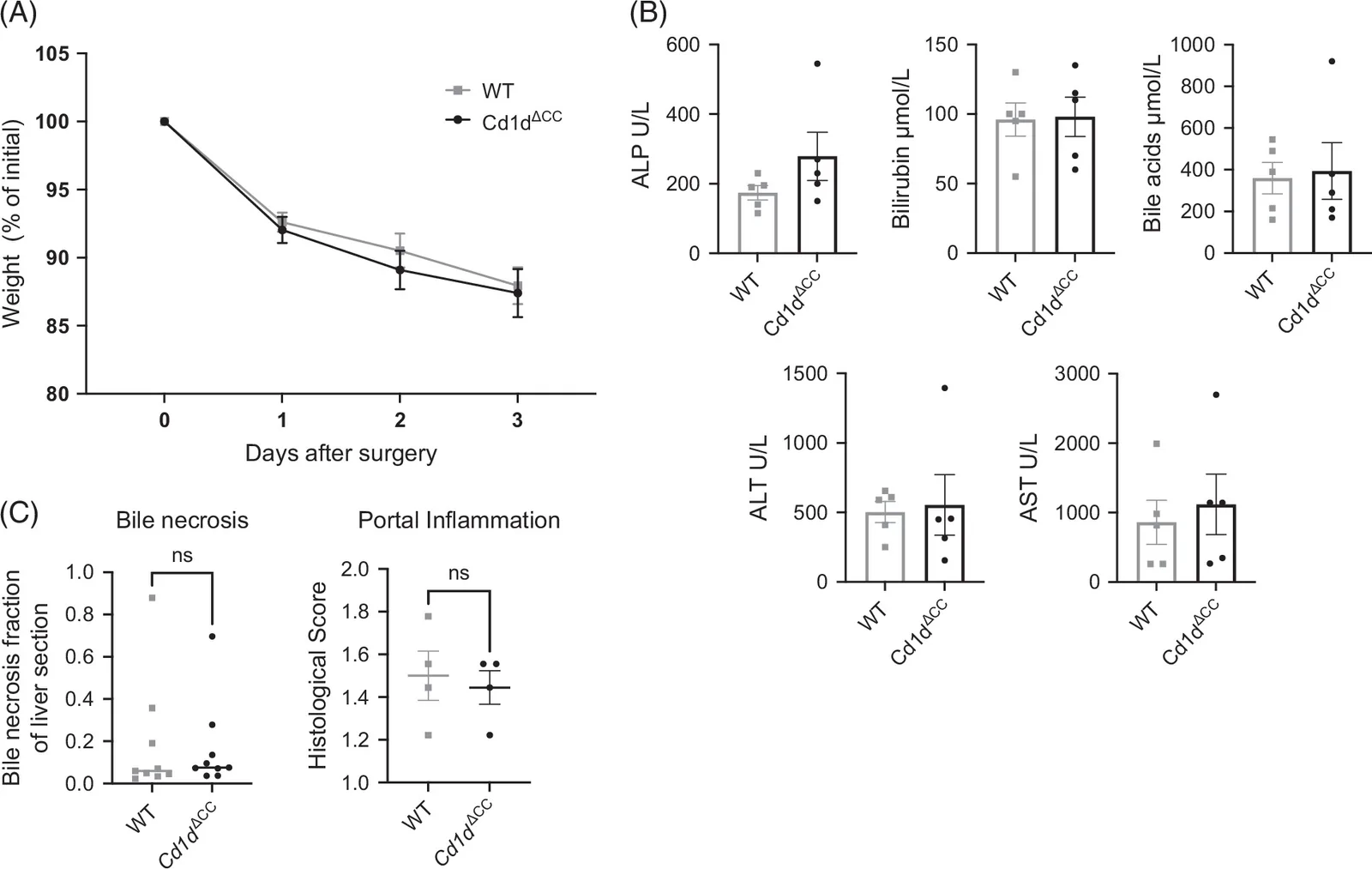
NKT cell–mediated pathology in bile duct–ligated mice is not driven by cholangiocytes. (A, B) Weight curve and serum biochemistry comparing WT (n=5) and *Cd1d* ^*ΔCC*^ (n=5) for 3 days after BDL. (C) Semiautomated quantification of the percentage of necrosis in WT and *Cd1d* ^*ΔCC*^ mice 3 days after BDL using an artificial neural network algorithm and assessment of portal inflammation in WT and *Cd1d* ^*ΔCC*^ mice 3 days after BDL by using a predefined histology score from 1 to 3. All experiments were repeated 6 times independently, and results from one representative experiment are shown as mean ± SEM. Statistical significance was evaluated by a 2-tailed Student *t* test. Abbreviations: BDL, bile duct ligation; WT, wild type.

## DISCUSSION

The pathogenic process in cholestasis has a clear contribution of the immune system,^2^ and in this study, we demonstrate that in response to the accumulation of bile in the liver of bile duct–ligated mice, hepatic and circulating iNKT cells are specifically activated and participate in disease propagation. Balancing tolerability and protection against liver injury is essential to control hepatic disease processes, and NKT cells are important mediators of the breach of tolerance with their on-site location in the sinusoids and rapid cytokine release.^33^^,^^34^ T-cell immune infiltrates are found around the bile ducts in cholestatic liver diseases such as PBC and PSC, and in PBC, these infiltrates are enriched in iNKT cells,^35^ supporting the relevance of NKT cells in bile duct inflammation. Our experiments demonstrated a clear and exclusive activation of iNKT cells as compared to the remaining T cells, at day 3, supporting a role as an early initiator in the inflammatory response, and this selective activation persisted until day 5 after BDL. The absolute count of hepatic iNKT cells was reduced both at day 3 and at day 5 after BDL, which may be interpreted as an indirect sign of activation with downregulation of the TCR rather than an actual diminution of the population.^27^

We did not determine the nature of the antigens or other compounds activating the iNKT cells in this project, but bile is rich in lipids, and we have previously demonstrated that human bile contains a diverse set of lipids shown to activate both niNKT cells and iNKT cells,^14^^,^^32^ which might represent one of the activating mechanisms. Importantly, with the sinusoidal crawling of NKT cells under normal conditions, they may be directly exposed to bile in the setting of biliary leakages from the bile canaliculi during BDL.^5^ The activation of iNKT cells during cholestasis may also be a result of cytokines produced by activated or necrotic hepatocytes or nonbile-related endogenous lipids presented by CD1d-expressing APCs.^36^ An increased influx of neutrophil granulocytes and natural killer cells was detected at day 3 and 5, which is in line with the literature and, for neutrophil granulocytes, anticipated since their presence has been described as early as 8 hours after BDL, promoting hepatic injury.^37^

Despite technological advancement, including methods such as snRNA-seq applied in this project, detailed information on niNKT cells remains fragmented.^38^ In our experiments, we observed that iNKT cells were not the main drivers of disease since the phenotype in bile duct–ligated *Traj18* ^*−/−*^ mice was similar as in WT mice and in contrast to the protection in *Cd1d* ^*−/−*^ mice lacking both iNKT cells and niNKT cells. This opposes previous BDL findings^10^^,^^20^ utilizing a different *Traj18* ^*−/−*^ mouse model.^39^ However, it has since the initial publication been reported that this *Traj18* ^*−/−*^ mouse model, which is different from the one we use here, has, in addition to iNKT cell deficiency, a perturbated T-cell repertoire and lacks MAIT cells, which warrants caution in the interpretation of results from experiments using these mice.^28^^,^^40^ The divergent results could also be due to the different C57BL/6 background, as these mice were generated on the C57BL/6J background, while the *Traj18* ^*−/−*^ mice used in the present study were generated on C57BL/6NJ background previously shown to affect immune responses.^41^ The activated NKT cells in our experiments clearly have a pathogenic role with a milder clinical and biochemical phenotype and livers with less bile infarcts in *Cd1d* ^*−/−*^ mice. In murine liver immunopathology, iNKT cells are, in general, found to be proinflammatory, whereas sulfatide-reactive niNKT cells are anti-inflammatory,^42^ which contrasts with our observations. With snRNA-seq, we could describe the NKT cell–related cytokine profiles, including IFNγ expression, giving clues on driving factors. This phenotype is also supported by human data, where niNKT cells play a proinflammatory role in patients with autoimmune hepatitis and findings from a mouse model, where upregulation of CD1d on T cells leads to liver damage mediated through niNKT cells.^43^^,^^44^ Also, in inflammatory bowel disease, niNKT cells are established as proinflammatory in both murine models and in patients with ulcerative colitis.^45^ Furthermore, a sulfatide-reactive fraction of niNKT cells induced iNKT cell anergy through upregulation of CD1d on plasmacytoid dendritic cells.^46^

To investigate the potential human relevance of our murine findings, we analyzed an snRNA-seq dataset derived from explanted human livers and detected activated NKT cells in patients with PSC compared to disease controls. This points toward a role for NKT cells in human cholestatic disease and warrants further investigations.

To specifically interrogate the role of cholangiocytes in NKT cell–driven immunopathology during cholestasis, we created a new mouse model lacking CD1d on the biliary epithelium. CD1d on cholangiocytes is found to correlate with disease stage, being upregulated in early stages of PBC, whereas it is downregulated in later disease stages of PBC, PSC, and alcohol-associated disease,^12^ suggesting an involvement in the immunopathophysiology. We observed no differences in weight loss, liver biochemical or histological phenotype in *Cd1d* ^*ΔCC*^ mice compared to control mice following BDL. These results conclusively demonstrate that CD1d on cholangiocytes is not one of the main drivers of the pathological activation of NKT cells in bile duct–ligated mice. Other cell types, such as sinusoidal endothelial cells, hepatic stellate cells, Kupffer cells, dendritic cells, and hepatocytes, which all express CD1d, can likely play this role.^12^^,^^47^^,^^48^ However, the activation observed can also be CD1d-independent as NKT cells can respond to interleukins alone or in different combinations,^49^ chemokines and PAMPs.^50^

The novel *Cd1d* ^*ΔCC*^ model will be of interest for interrogations of other NKT-driven pathologies and may furthermore be adopted to interrogate the role of MR1 and MAIT cells, another important unconventional T cell in biliary pathology by crossing with another floxed model.

In conclusion, NKT cells are pathogenic drivers in the acute and intermediate phase of cholestasis with an interplay between niNKT cells and iNKT cells independent of cholangiocyte-mediated CD1d function. Despite their low frequency, these results inspire further exploration of the human NKT cell population in cholestatic liver diseases and highlight the utility of creating a cholangiocyte-specific *Cd1d* knockout model for understanding the interplay of NKT cells and cholangiocytes.

## Supplementary Material
