# Supplementary material for: Natural Killer T cells promote cholestatic liver disease independent of antigen presentation by cholangiocytes
Source: Hepatol Commun. 2026 Jan 16;10(2):e0862. doi: 10.1097/HC9.0000000000000862 (PMC13671599; doi:10.1097/HC9.0000000000000862)
Supplement: Supplementary file 1 [file hc9-10-e0862-s001.docx]

**Natural Killer T cells promote cholestatic liver disease independent of antigen presentation by cholangiocytes**

Laura Valestrand^1,2,3,4^, Kathrine S. Nordhus^1,2,3^, Thierry Claudel^5^, Jonas Øgaard^1,2,3^, Anna Frank^1,2,3,6^, Enya Amundsen-Isaksen^1,2,3,6^, Claudia Fuchs^6^, Natalie L. Berntsen^1,2,3^, Brian K. Chung^1,2,3^, Xiaojun Jiang^1,2^, Michael Trauner^5^, Espen Melum^1,2,3,4,6^

Table of Contents

Supplementary methods	2

Co-culture experiments with NKT cells.......................................................................2

Generation of *ASBTcre^+/-^* mice ......................................................................................3

Generation and breeding of *Cd1d^ΔCC^* mice ...................................................................3

Genotyping.....................................................................................................................3

Histology and scoring ...................................................................................................4

Human samples..............................................................................................................5 Immunohistochemistry .................................................................................................5 Immunofluorescent staining ..........................................................................................6

Mice................................................................................................................................7

Mouse tissue collection and extraction of primary lymphocytes...................................7

Organoid cell culture......................................................................................................8

Single-nuclei RNA-sequencing of formalin-fixed paraffin-embedded liver blocks .....8

**Supplementary figures, tables and legends** .............................................................10

Supplementary figure 1................................................................................................10

Supplementary figure 2 ...............................................................................................12

Supplementary figure 3................................................................................................13

Supplementary figure 4................................................................................................14

Supplementary table.....................................................................................................15

Supplementary references............................................................................................15

# Supplementary methods

*Co-culture experiments with NKT cells*

After attaching the smaller cholangiocyets clumps to the collagen layer on the surface of the culture plates, the cells were washed twice with DMEM and then cultured with a synthetic analogue (*i.e.* KRN 7000, Avanti polar lipids, Alabaster, AL, USA) of the potent iNKT cell activating ligand α-galactosylceramide (α-GalCer). The cells were incubated overnight and then washed three times with DMEM to remove remaining unbound α-GalCer. A previously described NKT-cell hybridoma cell line (DN32.D3)^1^ was added (100,000 cells/well) to the cholangiocytes and co-cultured for 19h. Co-culture experiments were repeated in two independent experiments, using two independently cultured organoid cell lines for *Cd1d^ΔCC^* derived organoids, and three for WT and *Cd1d^-/-^* derived organoids. Measurement of IL-2 secreted by activated NKT cell hybridomas, by ELISA (BD Biosciences, Franklin Lakes, NJ, USA) was performed on collected cell culture supernatant.

*Generation of ASBTcre^+/-^ mice*

Homologous recombination in *E. coli*^2^ was performed with a cassette containing a 5´ 150 bp homology arm to the *Asbt* gene, the improved Cre recombinase (iCre)^3^, a bovine growth hormone polyadenylation (polyA) signal, an ampicillin resistance gene flanked by two FRT sites, and second 3´ homology arm of 200 bp to the *Asbt* gene. The homology arms were designed to replace the endogenous *Asbt* ATG with the ATG of the iCre construct as previously described^4^. After homologous recombination, the ampicillin cassette present in the BAC, flanked by FRT sites, was removed upon electroporation of a Flp expressing plasmid [706-Flp^5^]. 50 μg of the modified *Asbt* iCre BAC digested with NotI was applied to a selfmade column filled with Sepharose CL-4b (Pharmacia) which had been pre-equilibrated with oocyte microinjection buffer (10 mM Tris/HCl pH 7.4, 0.1 mM EDTA, 100 mM NaCl) ^6^. Fractions of 0.5 ml were collected, and the BAC integrity was analyzed using pulsed field gel electrophoresis before pronuclear injection in C57BL/6-derived oocytes.

*Generation and breeding of Cd1d^ΔCC^* *mice*

For maintenance, *Cd1d^ΔCC^* mice were bred with *Cd1d1^fl/fl^* mice and genotyped at each round to confirm the heterozygous presence of the *cre* gene and homozygous presence of *loxP* sites, before experiments. In *Cd1d1^fl/fl^* mice, *loxP* sites are inserted at each side of the *Cd1d1* gene, the homologous *Cd1d2* gene will not be expressed at a protein level due to a frameshift mutation^7,8^.

*Genotyping of ASBTcre^+/-^*

The primers CTTTCTGTCTTGGCCAGTAGTTTCTCTGG and GCTTGCAGGTACAGGAGGTAG were used in a polymerase chain reaction (PCR) reaction with conditions as follows: 94°C for 5 min (step 1), 94°C for 30 sec (step 2), 60°C for 30 sec (step 3), 72°C for 2 min and 30 sec (step 4), step 2-4 repeated for 32 cycles, 72 °C for 5 min (step 5), 4°C until PCR products were further analyzed. The PCR products were run on a 2% agarose gel, 120V for 40 min.

*Genotyping of Cd1d1^fl/fl^ mice*

The following primers were used; AAT AGG ATG TAA AAT GAA AAT GTA TCC and GGG TCC ATT CCA GAT ACA AA. In a PCR reaction with conditions as follows: temperature 94°C for 2 min (step 1), 94°C for 20 sec (step 2), 65°C for 15 sec (step 3), step 2-3 repeated for 10 cycles, 68°C for 10 sec (step 4), 94°C for 15 sec (step 5), 60°C for 15 sec (step 6), step 4-6 repeated for 33 cycles, 72°C for 2 min and 10 sec (step 7), 4°C until PCR products were further analyzed. The PCR products were run on a 2% agarose gel, 120V for 25 min.

*Histology and scoring of portal inflammation*

Liver formalin-fixed paraffin-embedded tissue was cut (3 µm) and deparaffinized. For hematoxylin and eosin (H&E) staining, slides were stained for six minutes with hematoxylin, rinsed in water, put in ammonia water, rinsed again in water and stained for one minute with eosin. The slides were dehydrated in ethanol, cleared in xylene and mounted. The liver sections were scored in a blinded fashion using the following parameters: Portal inflammation (0-3) where the score indicates degree of immune cell infiltration as either absent (0), mild (1), moderate (2) or marked (3). Images were generated from an Eclipse E400 Microscope with a DS-Fi1 camera controlled by NIS-elements BR 3.1 software (Nikon, Tokyo, Japan). In assessing the grade of necrosis, QuPath v3.4.0^9^ was used with a custom-built plugin to interface with slide storage library to enable QuPaths native machine-learning on the resulting large-scale dataset. An average of three annotations were made for each segmentation class (background, necrotic and normal tissue) on each of the regions of interest that represented the overall variation of morphological phenotype among the samples. We employed all features of σ = 10, and all color- and texture-based features of σ = 5, at an image resolution of 3.52 μm per pixel. After training the random forest classifier, the entire sections of all samples were analyzed and the percentage of necrotic areas in each sample was determined using built-in functions in QuPath.

*Human samples*

Briefly, tissue sections collected within 20 mins of liver explantation and cryopreserved. SnRNA-seq was performed using Chromium Next GEM single cell v3.1 3’ kits (10x Genomics) and snRNA-seq transcripts were analyzed in RStudio using Seurat and in Loupe browser v.7.0.1. As no specific markers encompass all NKT cells to the best of our knowledge, we defined NKT cells as (*THEMIS^+^NCAM1*^+^*SLC4A10^-^* or *THEMIS^+^KLRB1*^+^*SLC4A10^-^*) as *THEMIS*^+^ was highly expressed in the T cell cluster and strongly overlapped with the conventional markers *CD3* and *TRA/TRCB* that are often used with *NCAM1* and *KLRB1* to detect NKT cells^10^ but that were weakly detected by snRNA-seq in our dataset. These markers are not exclusively specific for NKT cells, but will include all NKT cells and by excluding *SLC4A10*^+^ cells the majority of mucosal-associated invariant T (MAIT) cells are excluded^11,12^.

*Immunohistochemistry*

Immunohistochemical staining of CD3e and lymphocyte antigen 6 complex locus G6D (Ly6G) was performed. Detailed antibody information is provided in **Supplementary Table 1**. Sections (3 µm) of formalin fixed paraffin-embedded liver tissue were deparaffinized before antigen retrieval. The slides were heated at 98°C in pressure cooker for 15 min in high pH (pH 9) buffer (Agilent Technologies, Santa Clara, CA, USA) for CD3e or low pH (pH 6) buffer (Agilent Technologies) for Ly6G, followed by 10 min cooling in room temperature (RT). Endogenous peroxidase was quenched by incubation in 3% H_2_O2 in PBS for 20 min. To prevent unspecific antibody binding, sections were incubated with Rodent block M (Biocare Medical, Pacheco, CA, USA) for 20 min, then 2.5% ready-to-use normal horse (CD3e) or goat (Ly6G) serum (Vector Laboratories, Burlingame, CA, USA), or 0.5% BSA/0.1% Tween 20 (Sigma-Aldrich, Merck KGaA, Burlington, MA, USA). Then, the sections were incubated with primary monoclonal antibodies for 60 min in RT (see details in Supplementary Table 1), washed thoroughly and incubated for 30 min with secondary antibodies; anti-rabbit (CD3e) or anti-rat (Ly6G) immunoglobulin (Vector Laboratories). DAB Peroxidase Substrate Kit (Vector Laboratories) for 10 (CD3e) or 5 (Ly6G) min was used for visualization, counter staining with Hematoxylin QS (Vector 5 Laboratories). The slides were dehydrated in ethanol, cleared in Histo-Clear and mounted.

*Immunofluorescent staining*

Immunofluorescence staining was performed as described previously by Berntsen *et al*^13^. Sections of cryopreserved liver tissue (3 µm) were fixed in acetone for 10 min, air dried for another 10 min and incubated for 60 min in RT with the primary CK19 antibody (clone EP1580Y, Abcam, Cambridge, UK). The sections were then washed and incubated 60 min with the secondary antibody (Alexa Fluor 546 goat Anti-Rabbit immunoglobulin, Invitrogen, Thermo Fisher Scientific, Waltham, MA, USA) in RT, followed by another wash. The sections were co-stained with a 60 min incubation of a monoclonal CD1d monoclonal antibody (clone 1B1, eBioscience, San Diego, CA, USA), washed and incubated another 60 min in RT with the secondary antibody anti-rat mouse adsorbed HRP (Vector Laboratories), followed by another wash. Finally, the slides were incubated 10 min in RT with Tyramid signal amplification solution (AAT Bioquest, Inc., Pleasanton, CA, USA) diluted in 0.003% H_2_O_2_ before they were dipped in dH2O and mounted using SlowFade Gold Antifade Mountant with DAPI (Invitrogen). The slides were subsequently imaged using Axioscan.Z1 (Zeiss, Jena, Germany), preserving exposure times and illumination intensities across slides and genotypes. Images were extracted using Qupath^9^.

*Mice*

Specific pathogen-free male C57BL/6J mice (The Jackson Laboratory, Bar Harbor, ME, USA, stock nr. 000664), C57BL/6NJ mice (The Jackson Laboratory, stock nr. 005304), *Traj18^-/-^* (The Jackson Laboratory, stock nr. 0305024)^14^, *Cd1d1^fl/fl^* mice (C57BL/6-Cd1d1^tm1.1Aben^/J; The Jackson Laboratory, stock nr. 016929)^15^, *ASBTcre^+/-^* mice and *Cd1d^-/-^* mice^8^ mice were housed in a minimal disease unit at the animal facility at Oslo University Hospital Rikshospitalet, Oslo, Norway.

*Mouse tissue collection and extraction of primary lymphocytes*

Blood was collected by heart or transthoracic puncture and incubated for 1h at room temperature before 10 min 4°C centrifugation at 12 000 rpm. Serum was then collected and stored at -80°C before analysis of ALT, AST, ALP and bilirubin using an ADVIA 1800 (Siemens, Munich, Germany) at The Central Laboratory, Norwegian School of Veterinary Science. The liver was sectioned and longitudinal halves of the left lateral, right lateral and caudate lobes were fixed in 4% neutral buffered formalin solution (Sigma-Aldrich Corporation, Merck KGaA, St. Louis, MO, USA) for 24h before paraffin embedment for histological investigations. Livers and spleens collected for extraction of lymphocytes were collected on tubes with cold PBS.

Livers were then mashed and filtered through a 70 μm cell strainer (Thermo Fisher Scientific) and washed before the pellet was resuspended with 4 mL of 40% Percoll and overlayed 2 mL of 60% Percoll (Sigma-Aldrich, St.Louis, MO, USA). After a gradient spin lymphocytes were collected and finally washed before resuspension in flow buffer. Spleens were mashed and filtered through a 40 μm cell strainer (Thermo Fisher Scientific), washed before red blood cells were lysed with RBC Lysis Buffer (10X) (Bio- Legend, San Diego, CA, USA) for five min followed by a final wash before resuspension in flow buffer

*Organoid cell culture*

In brief the culture medium consisted of supplemented Williams medium (Williams E+)^16^ (Thermo Fisher Scientific) + EGF (cat. Nr: 2028-EG, Bio-Techne, Minneapolis, USA) + R-Spondin (cat. Nr: 4645-RS-250, Bio-Techne) + DKK-1 (cat. Nr: 5439-DK-100/CF, Bio-Techne). Medium exchange was performed every 48h and the organoid lines were split every 5-7 days depending on organoid quality and quantity.

*Single-nuclei RNA-sequencing of formalin-fixed paraffin-embedded liver blocks*

Processing for GEM-X Flex Gene Expression analysis was performed using multiplexing as described in the user guide (CG000787)^17^. Following sequencing, the samples were aligned using 10x Genomics Cell Ranger v8.2.0^18^. Data was pruned for ambient RNA using CellBender^19^, and Scanpy^20^ and its Rapids-based equivalent (rapids-singlecell)^21,22^ were used for efficient data processing, inspection and extraction downstream. Data was batch corrected and duplet reduced using scVI^23^ in order to stabilize variance across cells and genes while preserving biological variation^24^. Lastly Celltypist^25^ was used for transferring labels from a previously annotated dataset from Guilliams et al., al.^26^, and cluster-wise majority calls were kept. Variance-stabilized data were used to compute UMAP projections, whereas log10-transformed counts served as the primary input for quantitative analyses and visualizations.

# Supplementary figures

**Supplementary figure 1**

**Supplementary Figure 1. Selectively activated iNKT cells in spleen, three and five days after bile duct ligation.**

(**A**) Percentage of splenic lymphocytes and absolute numbers of iNKT and TCRb^+^iNKT^-^ cells from bile duct ligated mice (n=7) compared to sham mice (n=5), 3 days after ligation, with median fluorescence intensity (MFI) of the activation markers CD69 and CD25. (**B**) Similar bar graphs as in (**A**), 5 days after bile duct ligation in mice (n=5) compared to sham operated mice (n=5). All experiments were repeated two independent times and results are shown as mean ± standard error of the mean (s.e.m.). Statistical significance was evaluated by a two-tailed Student’s. ** P <0.05, ** P<0.01, *** P<0.001.*

**Supplementary Figure 2**

**Supplementary Figure 2. Clinical score**

(**A**) Assessment of the clinical condition of WT and Cd1d^−/−^ mice after bile duct ligation, using a predefined score scheme including evaluation of; appearance (0-3) behavior (0-3), weight (0-12) (not included in graph) and clinical signs (0-3). Repetition 1 shown in (**A**), 2 in (**B**) and 3 in (**C**). Statistical significance was evaluated by a two-tailed Student’s. ** P <0.05, ** P<0.01.*

**Supplementary Figure 3**

**Supplementary Figure 3. Cytokines and activation markers of hepatic NKT cells**

Characterization of cytokine expression and activation markers of hepatic NKT cells (*Klrb1c*^+^/*Cd3e*^+^) three days after bile duct ligation of the mouse models (*Traj18^-/-^*^,^ n=3) and WT (wild type, n=4)) by single-nuclei RNA-sequencing.

**Supplementary Figure 4**

100 μm

**A**

**B**

**Supplementary Figure 4. Characterization of hepatic expression of CD1d**

(**A**) Immunofluorescent staining of CD1d expression in murine livers from *Cd1d*^ΔCC^, *Cd1d1*^fl/fl^ (wild type) and *Cd1d*^-/-^ mice. DAPI, blue; CK19, green; CD1d, red. (**B**) The percentage of each cell type expressing at least one transcript of *Cd1d1* in WT and *Traj18*^-/-^ mice (n=7)*. cDC1s, conventional type 1 dendritic cells; cDC2s, conventional type 2 dendritic cells; NK cells, natural killer cells; pDCs, plasmacytoid dendritic cells.* Scale bar= 100μm.

**Supplementary table**

**Supplementary Table 1. Overview of antibodies used for flow cytometry, immunohistochemistry or immunofluorescent staining.**

**Supplementary references**

1.	Gumperz JE, Christopher R, Makowska A, et al. Murine CD1d-restricted T cell recognition of cellular lipids. *Immunity*. 2000;12(2):211-221. doi:10.1016/S1074-7613(00)80174-0

2.	Youming Z, Frank B, Joep M, Francis S. A new logic for DNA engineering using recombination in Escherichia coli. *Nat Genet*. 1998;20(2):123-128.

3.	Shimshek DR, Kim J, Hübner MR, et al. Codon-improved Cre recombinase (iCre) expression in the mouse. *Genes (United States)*. 2002;32(1):19-26. doi:10.1002/gene.10023

4.	Casanova E, Fehsenfeld S, Mantamadiotis T, et al. A CamKIIα iCre BAC allows brain-specific gene inactivation. *Genes (United States)*. 2001;31(1):37-42. doi:10.1002/gene.1078

5.	Buchholz F, Ringrose L, Angrand PO, Rossi F, Stewart AF. Different thermostabilities of FLP and Cre recombinases: Implications for applied site-specific recombination. *Nucleic Acids Res*. 1996;24(21):4256-4262. doi:10.1093/nar/24.21.4256

6.	Yang XW, Model P, Heintz N. Hon1ologous recon1bination based n1odification in Esherichia coli and gern1line transinission in transgenic n1ice of a Bacterial Artificial Chrotnson1E. *Nat Publ Gr*. 1997;49:859-865.

7.	Sundararaj S, Zhang J, Krovi SH, et al. Differing roles of CD1d2 and CD1d1 proteins in type I natural killer T cell development and function. *Proc Natl Acad Sci U S A*. 2018;115(6):E1204-E1213. doi:10.1073/pnas.1716669115

8.	Sonoda KH, Exley M, Snapper S, Balk SP, Stein-Streilein J. CD1-reactive natural killer T cells are required for development of systemic tolerance through an immune-privileged site. *J Exp Med*. 1999;190(9):1215-1225. doi:10.1084/jem.190.9.1215

9.	Bankhead P, Loughrey MB, Fernández JA, et al. QuPath: Open source software for digital pathology image analysis. *Sci Reports 2017 71*. 2017;7(1):1-7. doi:10.1038/s41598-017-17204-5

10.	MacParland SA, Liu JC, Ma X-Z, et al. Single cell RNA sequencing of human liver reveals distinct intrahepatic macrophage populations. *Nat Commun*. 2018;9(1):4383. doi:10.1038/s41467-018-06318-7

11.	Chung BK, Jordens MS, Øgaard J, et al. Multimodal transcriptomics identifies metallothionein as a novel pathway in primary sclerosing cholangitis. *Hepatology*. 2025. doi:10.1097/HEP.0000000000001432

12.	Karlsson M, Zhang C, Méar L, et al. A single-cell type transcriptomics map of human tissues. *Sci Adv*. 2021;7(31). doi:10.1126/sciadv.abh2169

13.	Berntsen NL, Fosby B, Tan C, et al. Natural killer T cells mediate inflammation in the bile ducts. *Mucosal Immunol*. 2018;11(6):1582-1590. doi:10.1038/S41385-018-0066-8

14.	Chandra S, Zhao M, Budelsky A, et al. A new mouse strain for the analysis of invariant NKT cell function. *Nat Immunol*. 2015;16(8):799-800. doi:10.1038/ni.3203

15.	Bai L, Deng S, Reboulet R, et al. Natural killer T (NKT)-B-cell interactions promote prolonged antibody responses and long-term memory to pneumococcal capsular polysaccharides. *Proc Natl Acad Sci U S A*. 2013;110(40):16097-16102. doi:10.1073/pnas.1303218110

16.	Tysoe OC, Justin AW, Brevini T, et al. Isolation and propagation of primary human cholangiocyte organoids for the generation of bioengineered biliary tissue. *Nat Protoc 2019 146*. 2019;14(6):1884-1925. doi:10.1038/s41596-019-0168-0

17.	Genomics. 10x. GEM-X Flex Gene Expression Reagent Kit for Multiplex samples. ttps://www.10xgenomics.com/support/flex-gene-expression/documentation/steps/library-prep/gem-x-flex-gene-expression-reagent-kit-for-multiplex-samples.

18.	Zheng GXY, Terry JM, Belgrader P, et al. Massively parallel digital transcriptional profiling of single cells. *Nat Commun*. 2017;8:14049. doi:10.1038/ncomms14049

19.	Fleming SJ, Chaffin MD, Arduini A, et al. Unsupervised removal of systematic background noise from droplet-based single-cell experiments using CellBender. *Nat Methods*. 2023;20(9):1323-1335. doi:10.1038/s41592-023-01943-7

20.	Wolf FA, Angerer P, Theis FJ. SCANPY: large-scale single-cell gene expression data analysis. *Genome Biol*. 2018;19(1):15. doi:10.1186/s13059-017-1382-0

21.	Dicks S, A. P, Pintar J, et al. scverse/rapids_singlecell: v0.10.6 (v0.10.6).Zendo. *zendo*. 2024.

22.	Vega J, Li M, Aguillera K, Goel N, Joshi E, Durica KC, Kunta AR LC. RAPIDS: Reproducible Analysis Pipeline for Data Streams Collected with Mobile Devices. *JMIR Prepr 18/08/202023246*. doi:10.2196/preprints.23246

23.	Gayoso A, Lopez R, Xing G, et al. A Python library for probabilistic analysis of single-cell omics data. *Nat Biotechnol*. 2022;40(2):163-166. doi:10.1038/s41587-021-01206-w

24.	Luecken MD, Büttner M, Chaichoompu K, et al. Benchmarking atlas-level data integration in single-cell genomics. *Nat Methods*. 2022;19(1):41-50. doi:10.1038/s41592-021-01336-8

25.	Domínguez Conde C, Xu C, Jarvis LB, et al. Cross-tissue immune cell analysis reveals tissue-specific features in humans. *Science*. 2022;376(6594):eabl5197. doi:10.1126/science.abl5197

26.	Guilliams M, Bonnardel J, Haest B, et al. Spatial proteogenomics reveals distinct and evolutionarily conserved hepatic macrophage niches. *Cell*. 2022;185(2):379-396.e38. doi:10.1016/j.cell.2021.12.018
